# 'It's their mouth at the end of the day': dental professionals' reactions to oral health education outcomes

**DOI:** 10.1038/s41415-022-4978-z

**Published:** 2022-09-22

**Authors:** Emma Barnes, Alison Bullock, Ivor G. Chestnutt

**Affiliations:** 4141510429001 School of Social Sciences, Cardiff University, Cardiff, UK; 4141510429002 School of Dentistry, Cardiff University, Cardiff, UK

## Abstract

**Introduction** Research has established varying levels of efficacy of oral health education (OHE) efforts. However, little is known regarding how outcomes impact dental professionals and their OHE practice. This study explores dental professionals' reactions to varying OHE outcomes and their motivations to persist with their efforts.

**Methods** Qualitative, semi-structured interviews were conducted with dental team members working in mainly NHS general dental practices in South Wales, UK. Interviews were conducted face-to-face pre-COVID-19 and then by telephone, transcribed and analysed thematically.

**Results** In total, 30 interviews were conducted (17 dentists, 6 dental therapists and 7 dental nurses). Pleasure was gained from improved patient oral health. Responses to non-adherence included disappointment, frustration and acceptance. Acceptance centred around a shared responsibility for oral care between clinician and patient and reassurance that they had 'done their job'. The unpredictability of patient adherence aided OHE motivation; efforts might eventually inspire patient action or might align with patient readiness to change.

**Conclusions** This study reveals how OHE outcomes impact on dental professionals' perceptions of their role and personal motivations for continued educational efforts with patients. Greater emphasis on both preventative dentistry and self-care, coupled with understanding of the complex factors influencing oral health behaviour, would aid motivation for OHE.

## Key points

- Oral health education (OHE) effectiveness is dependent on a complex relationship of individual and situational factors.
- The outcomes of OHE interactions can have an effective impact on dental professionals and potentially colour their view of future OHE attempts.
- Acceptance of unpredictable patient outcomes can help maintain motivation for OHE. Acceptance of patient agency and of shared responsibility can lessen feelings of frustration and disappointment in 'non-compliance'.

## Introduction

Re-orientation of dentistry towards prevention is leading to greater emphasis on attempts to encourage patient self-care through oral health education (OHE) in general dental practice.^1^ However, the factors that influence oral health behaviour, OHE interactions and their outcomes are complex.^2^^,^^3^^,^^4^^,^^5^^,^^6^When such complex factors are not reflected on by dental professionals, if patients do not follow advice, for whatever reason, it may lead to disappointment and scepticism for future attempts.^7^ This paper addresses the effect of patient outcomes following OHE on dental professionals.

OHE interventions typically address the lifestyle-related, common, oral health risk factors for dental caries, periodontal disease and oral cancer. OHE provides an opportunity for a conversation between the dental professional and the patient which aims to offer knowledge and change attitudes and behaviours.^8^ During this interaction, the patient can gain understanding of the preventable causes of oral diseases and the dental professional can discuss ways to modify factors in the patients' behaviours (for example, toothbrushing with fluoride toothpaste) or lifestyle (for example, smoking cessation, reducing alcohol intake, or reducing sugar in their diet) that may lead to oral disease. Both parties should then agree a mutually acceptable and practical pathway (for example, an amended cleaning regime, or referral to a smoking cessation programme) for the patient to follow.^9^ Opportunities for what Holiday *et al*.^10^ referred to as 'teachable moments' may arise during the dental examination, such as the identification of tooth staining or tooth loss, which provide an opportunity to discuss smoking cessation.

Positive patient outcomes following OHE have been identified as a source of personal satisfaction for dental professionals owing to a concern for their patients' best interests and also as a reassurance of their own skills and good practice.^11^ Conversely, frustration from poor outcomes following OHE activity have been noted to impact on some dental professionals' satisfaction with their work, their perceptions of OHE efficacy, the motivation to provide it and it's effectiveness.^12^^,^^13^^,^^14^^,^^15^ Negative outcomes may also influence dental professionals' perceptions of patients. Lack of patient interest, perceived or real, has been cited as a barrier to OHE for dental professionals.^11^^,^^13^^,^^14^^,^^16^^,^^17^^,^^18^^,^While patients who have previously displayed efforts to maintain their oral health were thought to be those who would benefit from preventive activity,^19^ 'unreliable' patients who were judged not to take responsibility for their own oral health were seen as frustrating and unlikely to benefit from prevention.^20^ However, other papers report dental professionals trying out different approaches at the next appointment with previously non-responsive patients.^13^

There is a relative dearth of literature on how general dental professionals view OHE, or their motivation in offering OHE to patients.^11^ With the increasing focus on OHE to encourage patient self-care in oral healthcare, it is important to further understand how dental professionals respond to varying patient outcomes and how it impacts them both personally and professionally. This research aimed to explore dental professionals' reactions to varying OHE outcomes and their motivations to persist with their efforts.

## Methods

Qualitative semi-structured interviews were conducted with dentists, dental therapists, dental hygienists and dental nurses working in mainly NHS general dental practices in two health boards in South Wales, UK. The interviews were conducted as part of a wider study on OHE provision in general dental practices.^2^

The study was conducted both before and after the onset of the COVID-19 pandemic. Prior to the COVID-19 restrictions (December 2019 to March 2020), case study dental teams were recruited through letters to the practice and semi-structured interviews with all members of the dental team were conducted face-to-face in the practices, either one-to-one or in together in small focus groups if requested (identified by the prefix 'CS' in quotations). Restrictions imposed by the pandemic during the data gathering period (May 2020 to August 2020) meant that semi-structured telephone interviews were conducted with individual dentists, dental hygienists and dental therapists (identified by the prefix 'Tele'). Participants were recruited through social media posts advertising the study and by telephoning dental practices. Informed consent was sought from each participant by providing an information sheet and the opportunity to ask questions before agreeing to the interview. This was carried out in person for the face-to-face interviews and via email and telephone for the later interviews. All interviews were audio-recorded, with participants' permission. Consent was reconfirmed immediately before the interview.

All audio files were transcribed verbatim and analysed using thematic analysis. Analysis followed the six-step procedure outlined by Braun and Clarke:^21^^,^^22^^,^^23^ familiarisation with the data; generating initial codes; generating themes; reviewing potential themes; defining and naming themes; and reporting. Coding was mostly conducted at the semantic level to provide a descriptive summary of recurrent patterns in the content relating to the research aim.^21^^,^^22^^,^^23^^,^^24^ Using NVivo 12,^25^ sections of text were then collated for each code and organised into initial themes. New understandings and associations were generated during the process of drafting the narrative and the themes were refined as required.

The study applied the Capability-Opportunity-Motivation-Behaviour (COM-B) model framework^26^ and the Theoretical Domains Framework (TDF)^27^ to better understand the influences on participants' professional behaviour. These frameworks have been used in studies of oral health interventions^28^^,^^29^^,^^30^ and are suggested to be useful in exploring healthcare professionals' engagement in opportunistic behaviour change interventions.^31^ COM-B has also been used in the recently published edition of the *Delivering better oral health* toolkit^32^ to explain the different factors that impede or facilitate patient behaviour change. COM-B refers to a 'behaviour system' comprising three factors that are seen as essential to generate behaviour: capability (C), opportunity (O) and motivation (M).^26^^,^^33^In other words, individuals need to be sufficiently capable to perform the behaviour, to have suitable opportunity and the motivation to do it. In the COM-B model, each component is broken down into two elements, with capability including psychological aspects, such as possession of the necessary knowledge and the ability to understand its application, and physical aspects, such as the skills to carry out the intended change.^26^^,^^29^^,^^30^ Motivation comprises reflective processes, such as planning and goal setting and automatic processes, such as the influence of habits and emotions.^26^^,^^29^^,^^31^ Opportunity factors are external to the individual and include physical factors, which are environmental, such as access to resources and materials, or social factors, which are social norms or behaviours that support or inhibit behaviour.^26^^,^^29^^,^^30^

Related to the COM-B model is the TDF.^27^^,^^34^Designed by a team of psychologists and health service researchers, the framework synthesises common elements from behaviour change theories into a series of 14 domains.^27^^,^^30^^,^^34^ These domains broadly map onto the three COM-B domains. For example, the domains 'knowledge', 'skills', and 'memory, attention and decision processes' all fall within the COM-B 'capability' domain. Domains such as 'social influence' and 'environmental context and resources' mirror the 'social' and 'physical' aspects of opportunity, respectively. The TDF domains were used alongside the COM-B to explore the influences within the capability, opportunity and motivation domains in more detail.^35^

Cardiff University acted as sponsor for this study (ref: SPON 1755-19) and HRA ethical approval was obtained (North West - Greater Manchester West Research Ethics Committee, ref: 19/NW/0568, 6 September 2019). Owing to changes to the protocol arising from COVID-19, two category C substantial amendments were approved by the sponsor and the two participating university health boards. These amendments related to the inclusion of remote recruitment and telephone interviewing of individual dental professionals (April 2020) and patients (October 2020).

## Results

### Participant characteristics

In total, 30 dental professional participants were interviewed (Table 1). Including trainees, these comprised 17 dentists (n = 3 in a focus group), seven dental nurses (DNs) (in two focus groups, n = 3/n = 4) and six dental therapists (DTs). Most participants had qualified in Wales (n = 17), were women (n = 21) and were working in practices taking part in the Wales contract reform pilot (n = 27). Also, 16 of the participants (six dentists, three dental therapists and seven dental nurses) were from two (case study) dental practices; the remaining 14 were from 13 different practices. The number of years qualified ranged from 1-32, with a mean of 12 and a mode of 8. Two dental nurses were still in training and two foundation dentists had qualified within the six months before the interview. Eight participants worked in dentist-/dental-nurse-only practices; one did not work alongside dental hygienists or dental therapists but had access to an oral health promoter within their practice. Participants talked of seeing a range of patients, with most reporting seeing NHS-exempt patients with high oral health care needs.

**Table 1:** Participant characteristics

**Characteristic**	**Number**
**Role**	
Principal dentist	6
Associate dentist	8
Foundation dentist	2
Dental therapist	6
Dental nurse	7
**Sex**	
Female	21
Male	9
**Years qualified**	
<1	1
1-5	5
11-15	1
16-20	1
21-25	5
26-30	2
31-35	1
6-10	12
In training	2
**Contract reform pilot practice**	
Yes	26
No	3

### Participants' knowledge and confidence in OHE

When talking about providing OHE, participants explained that they saw the aim of the task as providing information and tools that would enable patients to better care for their own oral health.

- '*Giving people the tools to be able to make good choices and look after their teeth, so hopefully they don't get problems in the future*' (Tele-03).

Alongside the provision of self-care advice, OHE activities aimed to change patient attitudes to encourage a sense of shared responsibility and self-efficacy in patients to help them to look after their own oral health.

Participants mostly reported being confident in their OHE knowledge and communication skills. Most participants reported doing '*quite a lot*' of training on both the clinical background of lifestyle risk factors and how to deliver OHE during their undergraduate or professional training. Reading guidelines and toolkits and professional journals were identified as examples of ways of keeping knowledge up-to-date. The requirement of continuing professional development (CPD) for maintaining professional registration also provided opportunities for learning. Participants indicated that although they may not gain much new clinical knowledge from engagement with OHE-specific learning activities, they may be introduced to ideas about different ways to communicate or motivate patients. Such knowledge was also gained from non-OHE courses and discussing OHE with colleagues provided insight and tips based on their professional experiences.

Good communication skills were characterised as the ability to tailor messages to different people and were a valued aspect of OHE. Both those with more OHE experience and those newer to OHE recognised that experience of communicating with patients was key to gaining confidence. Some felt less confident about their abilities during time-pressured appointments, where they spoke of concerns about accidentally omitting information that they would normally, or would have liked to, include. Others explained how they felt confident discussing topics that they had good knowledge about (for example, oral hygiene and diet) but were less confident about discussing non-clinical issues, such as new products or more lifestyle-related issues (for example, alcohol or substance abuse) if they had little training or experience. In these instances, they preferred to refer the patients to their general practitioner or another relevant service. Participants also expressed concern about patients viewing them as 'overstepping the mark' and going beyond their role by discussing lifestyle issues that may not have such an obvious association with oral health.

While most were confident in their communication skills in OHE, some acknowledged that this did not necessarily mean that their confidence extended to the likelihood of patients making changes as a result. Some explained that they often felt that the OHE interactions went well with patients who had appeared attentive and engaged in the interaction, but that this did not always translate into a positive patient outcome:

- '*So, I wouldn't say it's always successful. I feel confident in my ability to communicate but I don't always feel confident in the patient's ability to do*' (Tele-13, Dentist).

Participants related their concern over the unpredictability of patients' potential responses to different OHE topics to their confidence in that topic. Similarly, experience of unpredictable patient outcomes also impacted their confidence in the patient's role in the OHE interaction.

### Reactions to OHE outcomes and continued motivation

#### Gaining pleasure from improvement

Great pleasure was reported when patients follow advice and as a result improved their oral health. Examples were given of patients who had stopped smoking and those who had improved oral hygiene. These improvements were said to be most pleasing when they happened with patients who they did not expect would follow their advice:

- '*I thought nothing is going to happen and he came back a few weeks later and it was like he'd had nothing wrong with him at all. It was like it was a different person […] the difference was actually superb. One of the most pleasing results I've seen*' (Tele-7, Dentist).

It was also acknowledged that such 'successes' may not be a common occurrence in practice and so they are particularly pleasing when they happen:

- '*It's always nice when you do have that one person that does improve for the ten that didn't*' (Tele-08, Dentist).

#### Frustration and disappointment from lack of behaviour change

Lack of adoption of recommendations were said to sometimes lead to disappointment and frustration. These reactions arose from a feeling that they were wasting their time with OHE efforts and that patients were needlessly stuck in poor oral health. A description used by several participants was that they felt they were '*talking to a brick wall*' in their OHE efforts. Repeated efforts at OHE led to them feeling like they were giving the same information at every appointment with little to no impact on the patient, resulting in them sometimes feeling '*fatigued*', '*disheartened*', or '*dispirited*':

- '*It is a bit frustrating when they come and it's the same thing all the time. We can't really give them any other motivation than what we already give*' (CS2-01, DN).

Participants also explained how frustrating and sad it sometimes made them when they saw patients attending with poor oral health that could have been prevented or managed. Failure to make what they considered relatively small, basic changes, such as brushing their teeth regularly, or patients who were not progressing with their treatment because of lack of self-care, were noted as sources of frustration and disappointment:

- '*You're trying to help people and you know they're not listening and you have their best intentions at heart. Sometimes they come in and you see them and it can just break your heart*' (Tele-7, Dentist).

However, only one participant reported that these feelings impacted on the amount of time spent on OHE, and then only temporarily.

#### Acceptance and shared responsibility

Participants reflected that, with experience, they had grown to accept that many attempts at behaviour change will be successful. When they were newly qualified, some saw lack of adherence to advice or failure to change behaviour as a reflection of their own efforts or skills as their OHE training focused on how to communicate messages effectively. They explained that with experience, they came to accept that not all patients will be motivated or interested in making changes to improve their oral health:

- '*I'd just think, typical (laughs) […] but now I'm tough and I think well, there's going to be people that are going to listen and people that just don't and it's very difficult to change those [...] and then if they do, fantastic, give positive praise and all of that and if they don't, I think okay, well*...' (CS1-06, DT).

Another way to accept negative outcomes was to recognise that they had fulfiled their side of the patient-dental professional relationship but cannot control the patient once they leave their surgery. The knowledge that they had 'done their bit' by giving information and reinforcing the message helped them to accept that all efforts may not be successful:

- '*It's their mouth at the end of the day. So, you can't really feel anything towards it. You just try and help them as much as you can*' (CS1-05, DT).

This appraisal of the interaction emphasised the shared responsibility of patients in looking after their own oral health.

#### Unpredictable patient outcomes and getting it right at the right time

Despite the varying frequency of positive outcome from OHE efforts, participants said that they kept up OHE efforts with all patients, as the reasons for non-adherence vary for patients. OHE efforts were maintained with all patients because participants recognised that not all patients learn or retain information in the same way and repeated attempts allowed them to try to find another way to communicate the message more effectively. Participants also explained that some patients may have tried to make changes but failed to achieve or maintain them for some reason. In this situation, participants talked of working with the patient to find out why it failed and finding another approach. Also, they indicated that some patients may not be ready to make changes owing to other factors in their life but that circumstances may later change that make them amenable to making oral health improvements. With these patients, it was said to be a case of keeping on reinforcing the message until the time was right:

- '*Until they're willing to change or something else happens in their life that they decide to change, there's nothing you can do about it. You have to keep on telling them every time that they come in and just try and give them a nudge in the right direction*' (Tele-2, Dentist).

Opinions varied about whether they were able to predict when patients were going to make advised changes. Some reflected that they could usually tell which patients would make changes based on their level of interest and engagement in the discussion or with patients who had previously had the same information. Others explained how they had had positive discussions with patients who later show no improvement. Conversely, some had pleasant surprises when patients who had looked bored or shown little interest later made changes:

- '*I don't think you can really tell a lot of the time. I think you've just got to speak to each patient as an individual and then you don't really know what they'll do or what they won't do*' (CS1-03, Dentist).

Again, participants emphasised how important it was that they keep trying with all patients.

### Application of the COM-B and TDF framework

Participants' accounts were mapped on to the COM-B domains^26^^,^^33^ and the TDF domains.^27^ Table 2 provides a summary of the application of the COM-B and TDF frameworks.

**Table 2:** Application of COM-B and TDF to the dental professionals' engagement or otherwise in OHE

**COM-B domain**		**Application**
**Capability**	**Psychological**	Knowledge Undergraduate training, post-graduate, CPD, company representatives, personal experience and from discussion with peers. Recognition that patients may have had little education on oral health and so need to understand why it is important and how it can be maintained
		Skills Maintain OHE efforts using a range of approaches in case they find the appropriate way to motivate non-adherent patients
	**Physical**	Skills Capability to explain and demonstrate hygiene techniques
**Opportunity**	**Social**	Social influence OHE activity is greatly influenced by patient response. Activity is adapted according to patient response, for example, resistance or lack of interest. Recognition of patients' cultural attitudes towards oral health and expectations of oral health care
	**Physical**	Environmental context and resources Macro: shift to a preventive-focus in dentistry which is not supported by NHS funding Meso: high patient throughput and short appointment times. Impacts on staffing decisions for example, DT/DHs and upskilling DNs Micro: differing ways of fitting OHE in around the appointment time and influencing the content and delivery of the OHE messages. Acknowledged that some patients' personal and socioeconomic factors inhibit opportunity for oral health self-care. Maintenance of OHE efforts in case the patient's circumstances change
**Motivation**	**Reflective**	Social/professional role and identity Recognition that OHE is a key part of their professional role. Concern about patient perception of them overstepping their role and being intrusive
		Beliefs about capability Good knowledge and good confidence in their communication skills but the levels varied by topic for example, less confident about alcohol and smoking than oral hygiene and diet. Confidence based on training and experience
		Optimism Belief that dentistry is changing towards prevention. Unpredictability of patient adherence. Confident in their own skills but less confident in the patients' ability or willingness to change
		Beliefs about consequences Unpredictability of patient adherence. All noted that some patients had surprised them. Accepted not all patients would make changes but saw unpredictability as a reason to keep providing OHE to all
		Intentions Perceived that patients attend the practice with different expectations of dental care and their own role in maintaining their oral health
		Goals Saw the aim of OHE as to change patient expectations of dental care away from treatment-based, to encourage a shared responsibility for oral health
	**Automatic**	Social/professional role and Identity Teamwork used to share the workload and reinforce OHE messages. Optimising the training and skills of different roles
		Reinforcement Lack of financial incentive from remuneration. Pleasure from patient improvement. Comment on oral hygiene efforts to reinforce messages
		Emotion Pleasure gained from improved patient oral health. Disappointment and frustration from lack of change following OHE efforts. Acceptance of variation in outcomes, based on adoption of a shared responsibility with patients.

## Discussion

This research aimed to explore views on provision of OHE and changes made to behaviour after OHE. Interviews provide participant accounts of the topic, that is, what people say about a topic rather than necessarily objective reports about behaviour.^36^ Therefore, in this study, behaviours and actions that were reported are accounts, rather than necessarily an accurate reflection of the participants' OHE provision. Lack of generalisability to other participant groups or contexts is also a common criticism of qualitative research. However, this study intended to explore subjective understandings and experiences of OHE and perceived roles and responsibilities, rather than an objective study of its delivery and effectiveness. The generalisability of the findings to all dental professionals was not the intended outcome; rather, it is anticipated. However, this does not mean that the findings will not resonate with many clinicians, or provide insight and reassurance about their own clinical experience.^37^^,^^38^

While the COM-B and TDF frameworks are widely used in healthcare research, they are not without issue. Their broad, generic content is praised for its completeness but may also infer an inaccurate perception of simplicity.^39^ Some researchers are not rigorous in their application of the frameworks and instead are selective, only exploring the domains they perceive as relevant for their phenomenon.^30^ Despite drawing on behaviour change theories, both COM-B and TDF, like other behaviour change taxonomies, are descriptive frameworks rather than theories and do not explain the mechanisms operating between domains. It is thus not possible to conclude testable hypotheses of behaviour.^40^ Ogden^39^ points out that the 'gaps' in such frameworks do not account for variability and flexibility, stating 'the need for flexibility, variability and change according to not the type of behaviour, or the type of intervention or even the type of patient but how that individual…happens to feel, think, look, behave or respond at any particular time.'

Recognising these limitations, Teixeira^41^ recommends that researchers' 'efforts to synthesise and integrate information must be balanced with preserving depth, detail and diversity'.

The importance of a good relationship with patients has been noted to make the OHE discussion easier.^20^^,^^42^ Positive patient outcomes following OHE were identified as a source of satisfaction, even if it was acknowledged that such outcomes were infrequent. Dental professionals' opinions on whether they could predict which patients would follow advice varied but all noted that they had been surprised by some patients and that this was a motivator for continuing OHE efforts (motivation - beliefs about consequences). Kelly and Barker^43^ point out that without knowing the individuals' relationships and social influences, it is very difficult to predict how a specific patient will behave. Frustration and negative feelings resulting from patients' lack of behaviour changes discussed in this study also align with the literature.^12^^,^^13^ However, other findings suggesting that such responses may negatively influence dental professionals' enjoyment and belief in the efficacy of OHE, impeding future OHE efforts^15^ were not borne out in the interviews. Only one participant indicated that they felt a temporary reluctance to engage in OHE following lack of adherence (motivation - emotion, and optimism). Leggett and Csikar^14^ found that lack of patient change was demotivating for their dentist participants but they felt a professional responsibility to repeat OHE messages, while emphasising the patients' need for more responsibility. In this study, seeing the relationship as a shared responsibility and accepting that patients' oral health outside the practice was out of their control were also adopted as ways of building a rapport with patients but keeping a distance from the outcomes (motivation - emotion). Instead, participants retained control over their OHE provision, ensuring that they had 'done their job' by providing the patient with the appropriate advice to help them manage their own oral health (capability - knowledge; motivation - social/professional role and identity).

Dental professionals' explanations for continuing OHE attempts with previously non-compliant patients reflected two approaches. Some participants explained how they kept up attempts to communicate advice to patients in the hope that at some point they may communicate it in such a way that it would resonate with the patient and inspire them to make changes. With this explanation, the dental professionals put the emphasis on their OHE skills and finding a better way to communicate it to the patient (capability - skills; motivation - beliefs about capability; and optimism). Jensen *et al*.^11^ found that their participants emphasised the importance of patients' responsibility for their own oral health but also spoke of their own responsibility for providing information and the outcome. This position appears at odds with the shared responsibility approach and a desire for patients to take control of their self-care.

Other participants spoke of keeping it going until the patient was ready to make changes. This puts the emphasis on the patient's motivation and having change-supporting circumstances (opportunity - environmental context and resources). The OHE advice was intended to encourage a sense of self-efficacy and to dispel fatalistic approaches towards having a sense of no control over their oral health. Encouraging self-efficacy is in line with the drives towards preventive dentistry and policy.^44^^,^^45^ However, Garthwaite and Bambra^46^ caution that what some professionals 'see as fatalism or a low locus of control are revealed as realistic assessments of the limited opportunities people have to control their lives'. Dental professionals in this study were found to vary their approach to patients and recognised the competing priorities that some of their patients lived with, which lessened feelings of frustration or disappointment in patient non-compliance. Rather than these being simple cases of active non-compliance, the desire to comply may be strong but constrained by competing demands.

Participants spoke of their frustration when some patients were not interested in following advice owing to their attitudes towards oral health (opportunity - social influence) and their expectations of their engagement with dental services and their own oral health behaviours (motivation - intention). This reflects an expectation that patients should follow their advice but doesn't consider the fact that some patients may actively choose not to. Patients may appraise the perceived potential benefits relative to the potential costs involved before making any changes to their behaviour or lifestyle (reflecting patients' motivation - optimism and belief in consequences). The General Dental Council's *Standards for the dental team* state that dental professionals should 'recognise and promote patients' responsibility for making decisions about their bodies, their priorities and their care'.^47^ Such an approach is central to a person-centred care model of dentistry,^48^ where a patient is both a recipient of care and also a partner in determining the nature of the care to be provided.^49^ Participants talked of the importance of encouraging a sense of shared responsibility with patients but their accounts still reflect a paternalistic biomedical model of dental care,^48^ with the emphasis on conveying the 'correct' information with the expectation that the patient should follow the advice. While their recommendations may have obvious and real clinical relevance for the dental professional, they may not be viewed this way by the patient for who 'health' is an individual interpretation.^48^ These findings align with previous research that reports that, even when aiming for co-designed treatment plans, dental professionals still lead by offering patients a range of options that they considered to be in the patients' best interests and what the patient 'needs'.^50^

Stressors within the dental practice, such as the context of working within NHS dentistry, staffing and the dentist-patient relationship, can impact on dental professionals' overall stress levels.^51^ While good patient relationships are one of the predictors of job satisfaction,^52^ other aspects of challenging patient relationships have been shown to have greater impact on dentists' job satisfaction than non-compliance.^53^ However, minimising the negative emotional impact of OHE benefits the dental professional and their patients. Unlike restorative care, behavioural preventive changes may be difficult to measure or only be achieved by a small percentage of patients, leading to recommendations of adoption of a wider practice or public health definition of success than solely on an individual case basis.^7^^,^^54^ Training in working with patients from different backgrounds and helping dental professionals to appreciate the different factors which influence patients' behaviours was also recommended to help dental professionals adopt realistic expectations of patients' scope for change.^55^

## Conclusion

While the operative side of dentistry is relatively predictable and observable, the outcomes of preventive interventions are less tangible and more unpredictable. Prevention and OHE necessitate an acceptance of factors that are outside of the professional's control and requires a shift for some in how they view their role and responsibilities. Participants' engagement with OHE reflected aspects of all three COM-B domains and twelve of the TDF domains. The dental professionals in this study reported a good level of capability domain factors, such as knowledge and good confidence overall in their OHE skills and showed flexibility in approach responding to practical constraints in the appointment and their social interaction with the patient (social and physical opportunity). They were happy when patients' oral health had improved after being given advice, highlighting that it was why they do their job (motivation). For the same reason, lack of improvement following advice was a source of disappointment or frustration. Adoption of a sense of shared responsibility with the patient and acknowledging the boundaries of their role and professional influence fostered a sense of acceptance for unpredictable outcomes and helped maintain motivation to engage in future OHE attempts. However, some participants still held on to the idea that outcomes were dependent on their ability to communicate messages in the 'correct' way for the particular patient. Acknowledging the patients' agency in decision-making regarding their own oral health alongside their other capability, opportunity, and motivation factors can help dental professionals to further accept variations in outcomes and maintain motivation for OHE efforts.
